# PD-L1 and Immune Infiltration of m^6^A RNA Methylation Regulators and Its miRNA Regulators in Hepatocellular Carcinoma

**DOI:** 10.1155/2021/5516100

**Published:** 2021-05-15

**Authors:** Yingxue Lin, Yinhui Yao, Ying Wang, Lingdi Wang, Haipeng Cui

**Affiliations:** ^1^Department of Medicine, Affiliated Hospital of Chengde Medical University, Nanyingzi Road, Shuangqiao District, Chengde, Hebei, China; ^2^Department of Pathophysiology, Chengde Medical University, Anyuan Road, Shuangqiao District, Chengde, Hebei, China

## Abstract

**Background:**

The aim of this study was to systematically evaluate the relationship between the expression of m^6^A RNA methylation regulators and prognosis in HCC.

**Methods:**

We compared the expression of m^6^A methylation modulators and PD-L1 between HCC and normal in TCGA database. HCC samples were divided into two subtypes by consensus clustering of data from m^6^A RNA methylation regulators. The differences in PD-L1, immune infiltration, and prognosis between the two subtypes were further compared. The LASSO regression was used to build a risk score for m^6^A modulators. In addition, we identified miRNAs that regulate m^6^A regulators.

**Results:**

We found that fourteen m^6^A regulatory genes were significantly differentially expressed between HCC and normal. HCC samples were divided into two clusters. Of these, there are higher PD-L1 expression and poorer overall survival (OS) in cluster 1. There was a significant difference in immune cell infiltration between cluster 1 and cluster 2. Through the LASSO model, we obtained 12 m^6^A methylation regulators to construct a prognostic risk score. Compared with patients with a high-risk score, patients with a low-risk score had upregulated PD-L1 expression and worse prognosis. There was a significant correlation between risk score and tumor-infiltrating immune cells. Finally, we found that miR-142 may be the important regulator for m^6^A RNA methylation in HCC.

**Conclusion:**

Our results suggest that m^6^A RNA methylation modulators may affect the prognosis through PD-L1 and immune cell infiltration in HCC patients. In addition, the two clusters may be beneficial for prognostic stratification and improving immunotherapeutic efficacy.

## 1. Introduction

Hepatocellular carcinoma (HCC) is an increasingly serious health problem with nearly 600,000 newly diagnosed patients with liver cancer [1]. The most prominent features of HCC are invasiveness and frequent recurrence [2]. Although great progress has been made in the treatment methods in recent decades, the prognosis of HCC patients is not optimistic, and the 5-year survival rate is less than 20% [3]. Most patients with advanced HCC have a high rate of recurrence and metastasis after treatment, which may be one of the reasons for poor prognosis [4].

The occurrence of liver cancer is a complex process involving multiple risk factors. It mainly includes cirrhosis, alcoholism, diabetes, and metabolic syndrome [5]. Surgical resection and liver transplantation are common effective treatments [6]. However, as most patients with advanced disease are diagnosed, only 15% of HCC patients are likely to receive effective treatment [7]. Patients with early HCC are always asymptomatic or present with nonspecific symptoms, such as abdominal pain, jaundice, and weight loss, leading to HCC being initially undetected. Therefore, exploring the pathogenesis of hepatocellular carcinoma and searching for promising biomarkers for diagnosis and prognosis of hepatocellular carcinoma are helpful to provide effective therapeutic targets and improve the prognosis of patients.

N^6^-methyladenosine (m^6^A) is a ubiquitous internal modification of RNA at the posttranscriptional level [8]. It plays a key role in pre-RNA splicing, translation regulation, and RNA decay [9, 10]. Studies showed that abnormal m^6^A modification is closely related to the progression of hepatocellular carcinoma [11, 12].

The liver tissue can stimulate immune response and prevent undesirable pathogen attack and tumorigenesis [13]. Sinusoidal endothelial cells in the liver can express programmed cell death ligand-1 (PD-L1) of immunosuppressive molecules, thereby regulating the immunogenicity of the liver microenvironment [14]. Immunotherapy has been shown effective and safe in the treatment of large numbers of solid tumors, prolonging overall survival (OS) [15, 16]. Han et al. found that YTHDF1-deficient (YTHDF 1−/−) mice exhibited an antigen-specific elevation of antitumor response [17]. Therefore, m^6^A regulators involved in immune pathways may be considered a target to enhance the response of tumor immunotherapy [18].

It is well known that carcinogenesis is a multistep process that triggers the accumulation of genetic alteration [19]. Like other solid cancers, this also occurs in the development of HCC. With the advancement of HCC biology and molecular classification, it led to the discovery of different phenotypes of HCC and the discovery of significant molecular markers for treatment [20]. Therefore, molecular subtype-related features provide valuable information for treatment and prognosis. The aim of this study was to systematically evaluate the relationship between the expression of m^6^A RNA methylation regulators and prognosis in HCC.

## 2. Materials and Methods

### 2.1. Dataset

We obtained RNA-Seq transcriptome data and relevant clinical data from The Cancer Genome Atlas (TCGA) database for patients with primary hepatocellular carcinoma. This included 374 tumor specimens of hepatocellular carcinoma and 50 normal tissues. We downloaded transcript per million mapped read (FPKM) data which had been normalized by using Perl. All data are open; therefore, approval from the Ethics Committee is not required. GSE147889 included microRNA (miRNA) profiling of liver tissue specimens from ninety-seven samples of HCC tumor tissue, with corresponding samples of surrounding tissue.

### 2.2. Difference Analysis

The FTO, YTHDC2, YTHDC1, ZC3H13, METTL14, METTL3, HNRNPA2B1, HNRNPC, YTHDF1, METTL13, WTAP, RBM15, YTHDF2, ALKBH5, KIAA1429, and YTHDF3 are m^6^A methylation regulators. Differential expression analysis of m^6^A regulators and PD-L1 between HCC samples and normal was analyzed through the EdgeR R package [21]. A *P* < 0.05 was significant [22–24]. The immunoscore for each HCC patient was calculated through the estimate R package.

### 2.3. Consensus Clustering Analysis

The consensus clustering analysis of m^6^A RNA methylation regulators based on the expression in HCC samples was performed through the ConsensusClusterPlus package [25]. The Kaplan-Meier survival curves were plotted using survival R package. *P* < 0.05 was considered statistically significant. The Hallmark gene set for different HCC subtypes was analyzed through GSEA software. The enrichment pathway was determined by *P* value < 0.05 and NES.

### 2.4. Risk Score and Prognosis

Cox regression analysis and least absolute shrinkage and selection operator (LASSO) were performed to evaluate the impact of m^6^A methylation regulators on the prognosis of HCC, which is a regression analysis method that performs both variable selection and regularization in order to enhance the prediction accuracy and interpretability of the resulting statistical model. HCC samples were divided into different groups through the median risk score based on the coefficients of LASSO and the expression of m^6^A regulators.

## 3. Results

### 3.1. m^6^A RNA Methylation Regulator Levels in HCC

**Figure 1:**
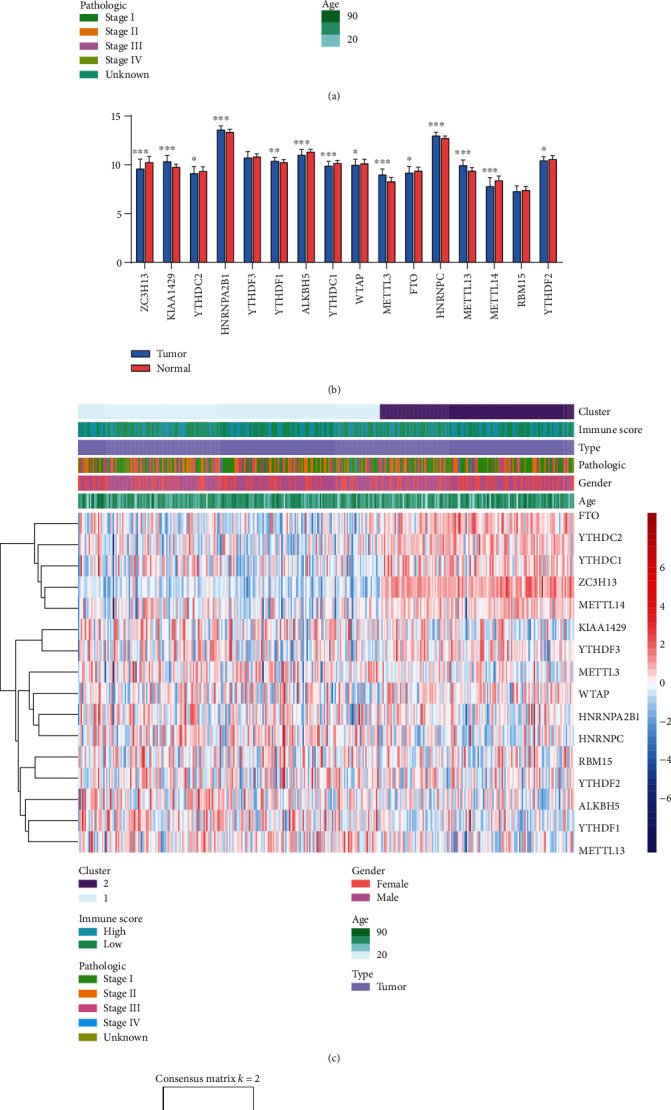
Differential features of m^6^A methylation regulators of HCC patients. (a) Expression of m^6^A RNA methylation regulators in HCC and normal. (b) Differential expression of m^6^A RNA methylation regulators in HCC and normal. (c) The *k* = 2 in consensus clustering matrix. (d) Heatmap for cluster 1 and cluster 2 samples of HCC patients. (e) Kaplan-Meier curves of overall survival in two clusters for HCC patients.

We compared the expression of sixteen m^6^A regulators between HCC and normal (Figure 1(a)). In addition to YTHDF3 and RBM15, the expression of these genes was significantly different in HCC compared with normal tissues (Figure 1(b)). This suggested that m^6^A RNA methylation regulators played an important role in the development of HCC.

*k* = 2 was determined based on consensus clustering analysis, and HCC samples were divided into two subtypes according to the expression of m^6^A methylation regulators, namely, cluster 1 and cluster 2 (Figure 1(c)). The expression of FTO, YTHDC2, YTHDC1, ZC3H13, and METTL14 in cluster 1 was significantly lower than that in cluster 2 (Figure 1(d)). The overall survival (OS) of cluster 2 was longer than that of cluster 1 (Figure 1(e)). The results showed that the subtypes of expression clustering in the m^6^A regulator were associated with the prognosis of HCC.

### 3.2. m^6^A RNA Methylation Affected PD-L1

**Figure 2:**
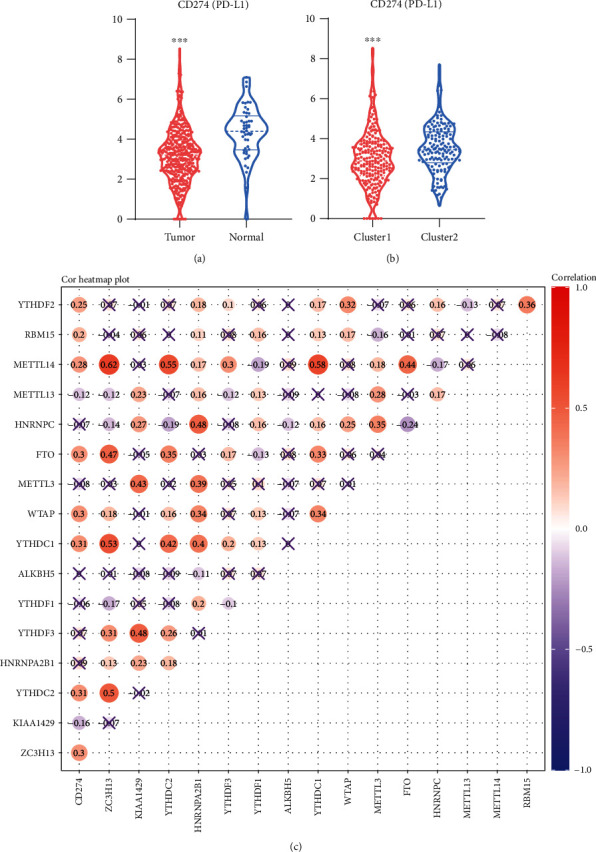
Expression level of PD-L1 was associated with m^6^A methylation. (a) Differential expression of PD-L1 between HCC and normal. (b) Differential expression of PD-L1 between cluster 1 and cluster 2. (c) Correlation between PD-L1 and m^6^A RNA methylation regulators. × represents *P* > 0.05.

We then evaluated the correlation between m^6^A methylation regulators and PD-L1. Compared with normal, the expression level of PD-L1 in HCC tissues was significantly higher (Figure 2(a)). As well as compared with cluster 2, PD-L1 was significantly higher in cluster 1 (Figure 2(b)). The results of correlation analysis showed that the expression of PD-L1 was positively correlated with the expression of METTL14, RBM15, YTHDF2, FTO, YTHDC1, WTAP, YTHDC2, and ZC3H13 and negatively correlated with the expression of METTL13 and KIAA1429 (Figure 2(c)).

### 3.3. Association of Immune Cell Infiltration with m^6^A RNA Methylation

**Figure 3:**
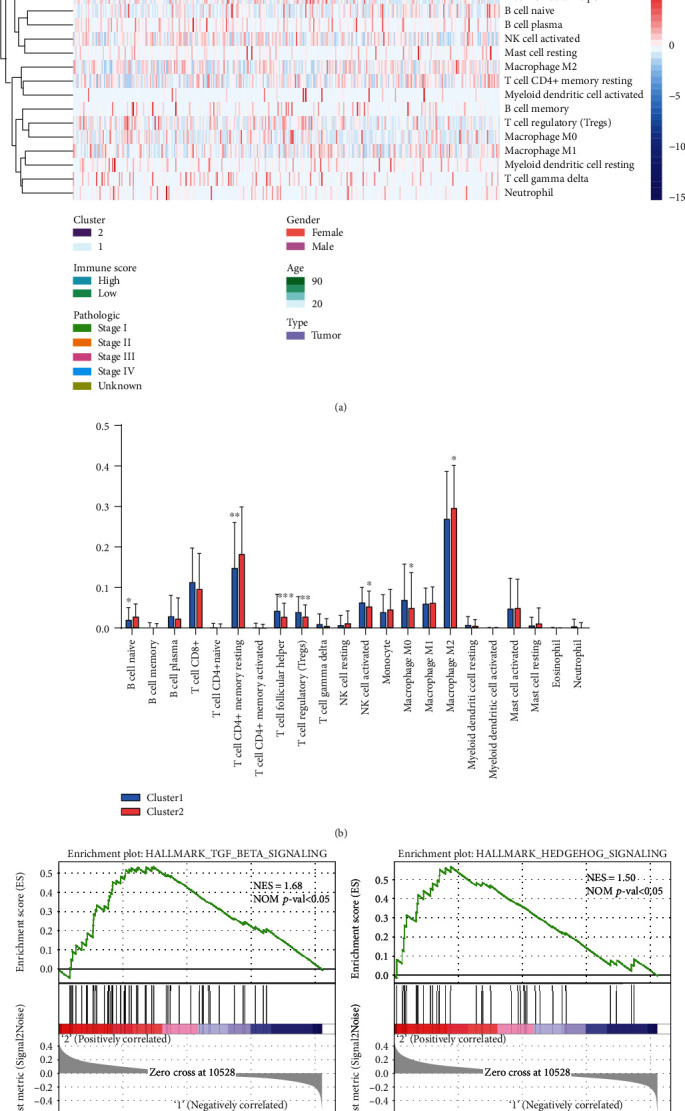
Immune cell infiltration in two clusters. (a) Infiltration level of immune cells in two clusters. (b) Differences of immune cell infiltration between cluster 1 and cluster 2. (c) GSEA showed that biological functions were differentially enriched in cluster 1 and cluster 2.

Next, we calculated the infiltration levels of 22 immune cell types between the two subgroups (Figure 3(a)). Cluster 1 showed higher levels of T cell follicular helper, NK cell activated, T cell regulatory (Tregs), and macrophage M0 (Figure 3(b)). Cluster 2 showed higher levels of B cell nave, T cell CD4+ memory resting, and macrophage M2. GSEA was used to elucidate the biological functional differences between the two subtypes. The results showed that TGF-beta signaling and Hedgehog signaling were dynamically correlated with cluster 2 (Figure 3(C)). Therefore, TGF-beta and Hedgehog signaling may be related to the tumor microenvironment of cluster 1/2.

### 3.4. Prognosis of HCC Affected by m^6^A RNA Methylation Regulators

**Figure 4:**
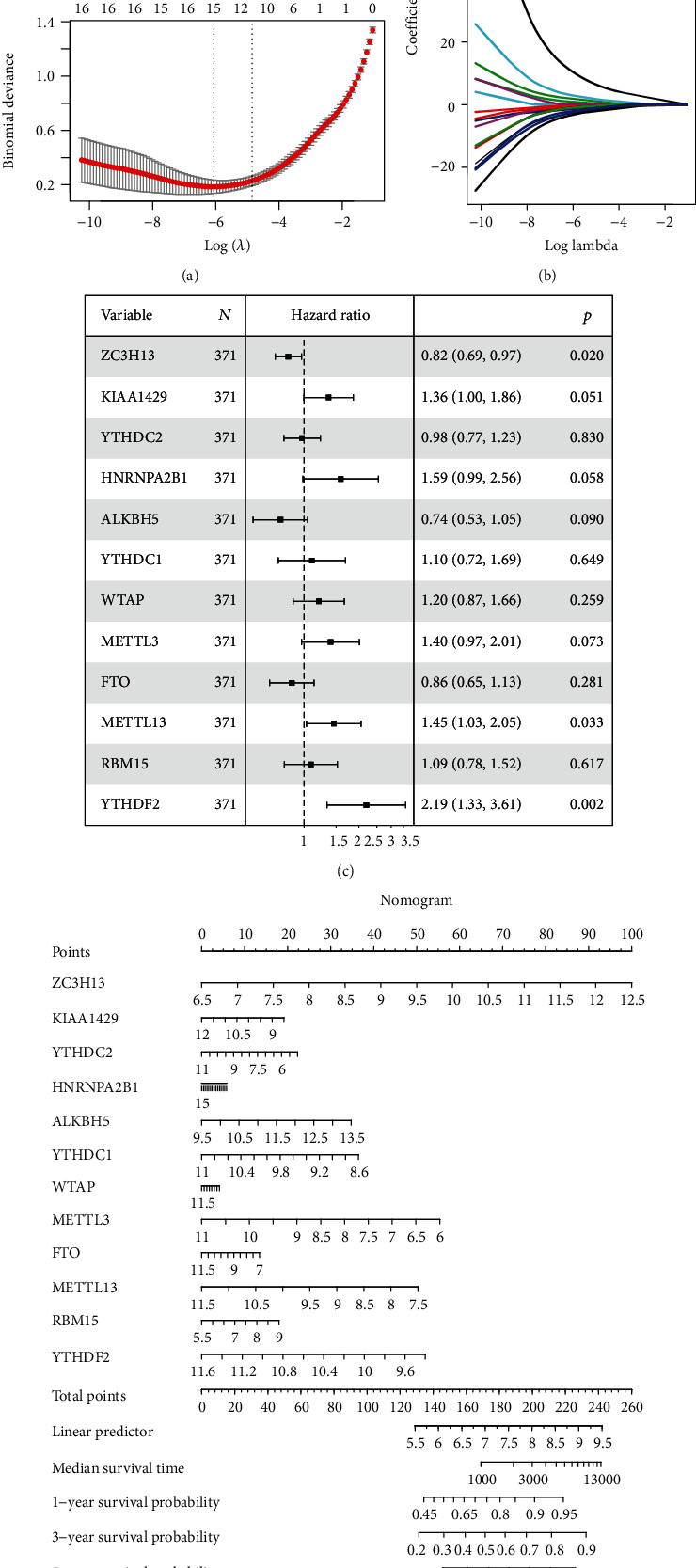
m^6^A methylation regulators influenced the prognosis of HCC. (a, b) LASSO regression analysis of m^6^A methylation regulators. (c) Forest map of twelve m^6^A methylation regulators predicting the impact on prognosis of HCC. (d) The nomogram to predict overall survival in HCC patients.

We performed LASSO regression analysis to identify the clinical significance of m^6^A regulators in HCC (Figures 4(a) and 4(b)). Twelve m^6^A regulators, namely, ZC3H13, KIAA1429, YTHDC2, HNRNPA2B1, ALKBH5, YTHDC1, WTAP, METTL3, FTO, METTL13, RBM15, and YTHDF2, were identified. Univariate Cox regression analysis showed that ZC3H13 may be a protective factor for HCC, while METTL13 and YTHDF2 are risk factors for HCC (Figure 4(c)). Nomogram results showed that increased expression of ZC3H13 was associated with longer OS of HCC (Figure 4(d)). Decreased expression of YTHDF2, METTL13, and METTL3 is beneficial to OS of HCC.

### 3.5. Risk Scores of m^6^A Methylation Regulators

**Figure 5:**
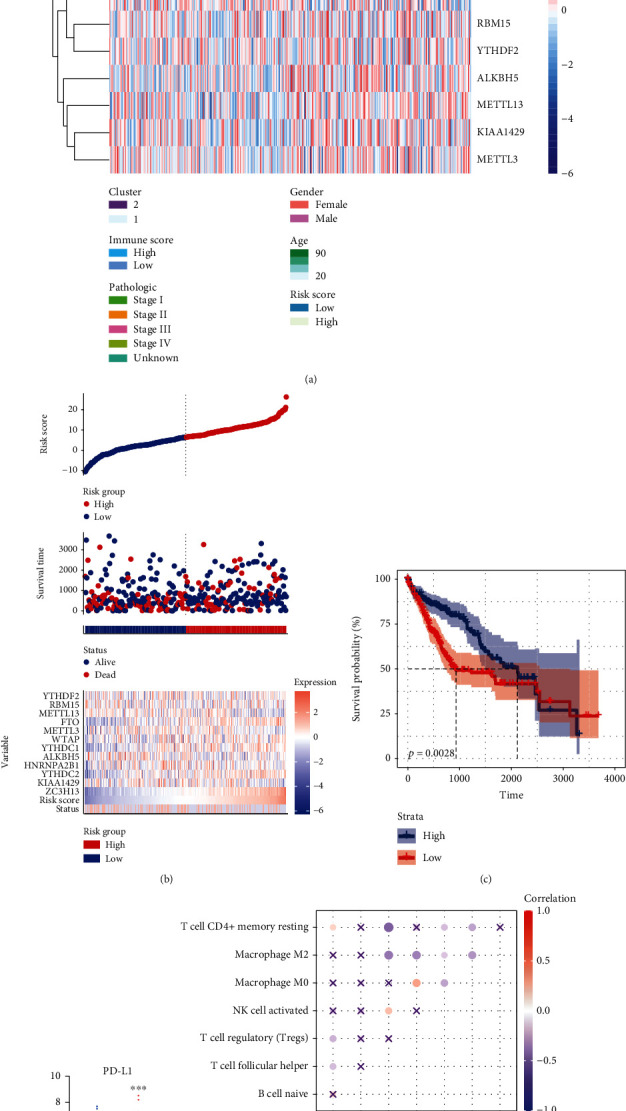
The role of risk score in the prognosis of HCC. (a) Heatmap of the high- and low-risk groups. (b) The risk score, OS, and heatmap of the twelve m^6^A regulator signatures. (c) Kaplan-Meier curves of OS based on the risk score for patients with HCC. (d) PD-L1 expression between the high- and low-risk groups. (e) Correlation between risk score and significant immune infiltrating cells.

The risk scores were calculated through the coefficients from the results of LASSO. Risk score = coefficients × expression of genes (5.9813 × ZC3H13 − 0.6146 × KIAA1429 + 0.3476 × YTHDC2 − 0.7669 × HNRNPA2B1 − 1.6292 × ALKBH5 − 0.1771 × YTHDC1 + 0.8649 × WTAP − 1.2779 × METTL3 + 1.6007 × FTO − 1.7691 × METTL13 − 0.2269 × RBM15 − 1.0176 × YTHDF2). The median risk score was used to divide HCC patients into the high-risk and low-risk groups (Figure 5(a)). Compared to the high-risk group, the levels of FTO, ZC3H13, YTHDC2, and YTHDC1 were lower in low risk. The mortality rate gradually decreased with the increase of the risk score (Figure 5(b)). The OS was longer, and the PD-L1 expression was lower in the high-risk group than in the low-risk group (Figures 5(c) and 5(d)). In addition, the risk score was significantly positively correlated with the level of T cell CD4+ memory resting and negatively correlated with infiltration level of Tregs and T cell follicular helper (Figure 5(e)). This result confirmed that the risk scores of m^6^A methylation regulator were associated with the immune microenvironment of HCC.

### 3.6. miRNAs Regulating m^6^A Regulators

**Figure 6:**
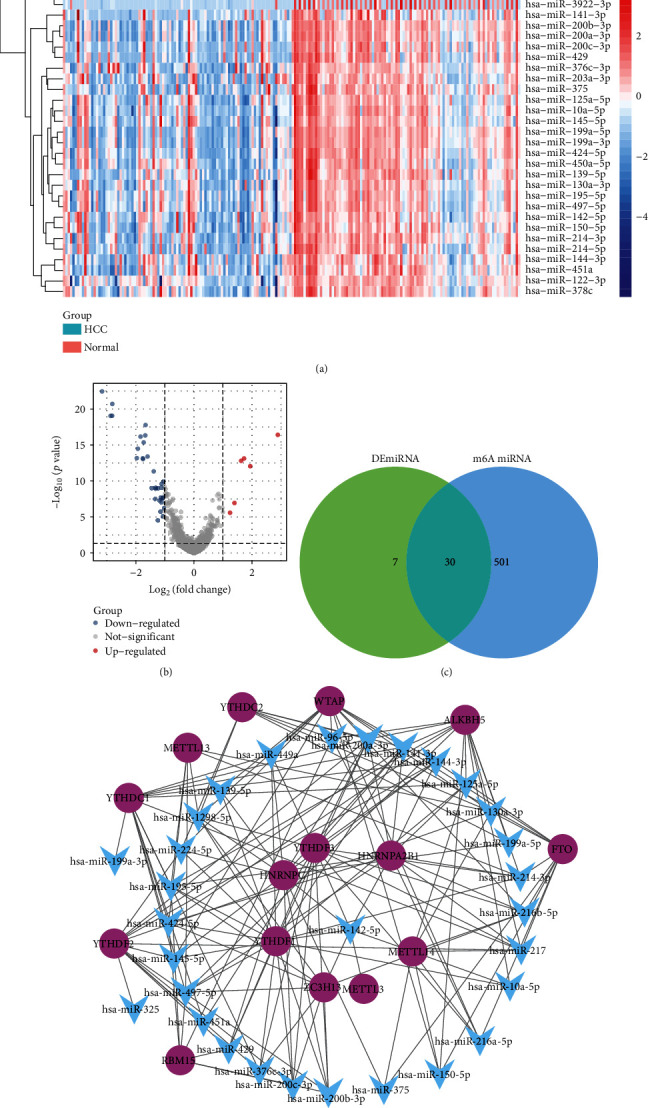
Regulatory network of miRNAs. (a) Heatmap of differential miRNA expression in GSE147889. (b) Volcano map of differential miRNA expression in GSE147889. (c) Intersection of differential miRNAs with predictive regulators of m^6^A regulator. (d) Regulation network of m^6^A regulators by HCC-related miRNAs.

By comparing differentially expressed miRNAs between HCC and control in GSE147889, we identified 37 miRNAs that may be involved in the regulation of HCC (Figures 6(a) and 6(b)). To identify the miRNAs that regulate m^6^A regulators, we predicted 531 miRNAs with targeted regulatory relationships through miRtarget. Among them, 30 miRNAs and differentially expressed miRNAs intersect and were considered regulators of m^6^A regulators related to HCC (Figure 6(c)), because hsa-miR-142-5p may be an important regulator as it regulated more m^6^A regulators (Figure 6(d)).

## 4. Discussion

The m^6^A methylation has many important biological functions and participates in the process of cancer [26, 27]. Herein, we found that the expression of m^6^A regulator is related to the prognosis of HCC and to the immune microenvironment. We have also attempted to elucidate some potential molecular mechanisms that may help make early diagnosis and develop molecular targeted therapies for hepatocellular carcinoma. In addition, we used the selected twelve m^6^A regulator to obtain a prognostic risk score.

At present, many evidences showed that m^6^A regulator is related to the progression of HCC [28]. In this study, compared with normal, YTHDF1 expression in HCC tissues was significantly increased, while YTHDF2 expression was significantly decreased. Elevated YTHDF1 promotes poor prognosis in HCC patients and is involved in regulating the metabolism and cell cycle progression of HCC cells [29]. A study reported that overexpression of YTHDF2 inhibits the proliferation and growth of HCC cells and promotes the apoptosis of HCC cells [30]. Studies have shown that YTHDC2 and METTL3 can promote the development of hepatocellular carcinoma [31, 32]. METTL13 promotes the growth and metastasis of HCC and is related to the survival status [33]. Consistent with our analysis results, the expression of METTL14 in HCC tissues was lower than that in normal tissues [34]. Contrary to our analysis, WTAP was significantly elevated in HCC, which was associated with poor survival outcomes [35]. ZC3H13 was downregulated in HCC and was a protective gene, which was also confirmed by other studies [36, 37]. The Cox risk score and clinical characteristic analysis confirmed that ZC3H13, METTL13, and YTHDF2 could be used as prognostic indicators and even as targets for new treatment of liver cancer.

Our results suggested that m^6^A methylation was associated with the development of hepatocellular carcinoma. OS and PD-L1 expression in cluster 2 was significantly different from that in cluster 1. The better prognosis of cluster 2 patients may be related to the lower expression level of PD-L1 [38]. GSEA results showed that TGF-*β* signaling and Hedgehog signaling were significantly enriched in cluster 2. Transforming growth factor beta (TGF-*β*) inhibits the occurrence of early HCC by inducing cell arrest and apoptosis but promotes the malignant progression of advanced HCC by promoting the survival, metastasis, migration, and invasion of tumor cells [39]. In HCC, aberrantly activated Hedgehog signaling promotes the development and invasion of HCC [40, 41]. This seems controversial with the good prognosis of patients with cluster 2. m^6^A methylation is complex in tumors, and its prognostic value in HCC needs further study.

In addition, the risk score was correlated with the expression of PD-L1. In the high-risk group, the PD-L1 expression was lower and had a good prognosis. Cluster 2 is in the high-risk score subtype of HCC. These findings suggested that m^6^A methylation regulation participated in the regulation of the immune microenvironment of HCC to some extent. Overexpression of miR-142 inhibits the invasion and angiogenesis of HCC cells and may be a potential therapeutic target for HCC [42]. Our results suggest that miR-142 may have a regulatory effect on m^6^A regulator. Interestingly, it was found that miR-142 was modified by methylation in tumor patients and cell lines and participated in the growth of HCC [43].

However, this study has some limitations. Firstly, our analytical data were from public databases; there may be some deviations. Secondly, more data, as well as large numbers of clinical and experimental data, are needed to verify the accuracy and reliability of our analysis results. In addition, whether the classification based on consistent clustering has clinical significance for HCC needs further confirmation.

## 5. Conclusions

In conclusion, the results suggested that the levels of m^6^A methylation regulator were related to the OS and immunity in HCC. This study has important proof value for demonstrating the impact of m^6^A methylation in HCC. Furthermore, ZC3H13, METTL13, and YTHDF2 may be potential predictors and therapeutic targets for HCC. miR-142 may regulate m^6^A methylation and participate in HCC.

## Data Availability

We obtained RNA-Seq transcriptome data and relevant clinical data from The Cancer Genome Atlas (TCGA) database for patients with primary hepatocellular carcinoma. GSE147889 included microRNA (miRNA) profiling of liver tissue specimens from ninety-seven samples of HCC tumor tissue, with corresponding samples of surrounding tissue.

## References

[1] Zhang J., Lou W. (2020). A key mRNA-miRNA-lncRNA competing endogenous RNA triple sub-network linked to diagnosis and prognosis of hepatocellular carcinoma. *Frontiers in Oncology*.

[2] Critelli R. M., De Maria N., Villa E. (2015). Biology of hepatocellular carcinoma. *Digestive Diseases*.

[3] Lou W., Liu J., Gao Y. (2018). MicroRNA regulation of liver cancer stem cells. *American Journal of Cancer Research*.

[4] Zhong J. H., Xiang X., Wang Y. Y. (2020). The lncRNA SNHG16 affects prognosis in hepatocellular carcinoma by regulating p62 expression. *Journal of Cellular Physiology*.

[5] Zhao X., Dou J., Cao J. (2020). Uncovering the potential differentially expressed miRNAs as diagnostic biomarkers for hepatocellular carcinoma based on machine learning in The Cancer Genome Atlas database. *Oncology Reports*.

[6] Liu H., Wang X., Feng B. (2018). Golgi phosphoprotein 3 (GOLPH3) promotes hepatocellular carcinoma progression by activating mTOR signaling pathway. *BMC Cancer*.

[7] Roxburgh P., Evans T. R. (2008). Systemic therapy of hepatocellular carcinoma: are we making progress?. *Advances in Therapy*.

[8] Fu Y., Dominissini D., Rechavi G., He C. (2014). Gene expression regulation mediated through reversible m^6^A RNA methylation. *Nature Reviews. Genetics*.

[9] Roignant J. Y., Soller M. (2017). m^6^A in mRNA: an ancient mechanism for fine-tuning gene expression. *Trends in Genetics*.

[10] Gu C., Shi X., Dai C. (2020). RNA m^6^A modification in cancers: molecular mechanisms and potential clinical applications. *The Innovation.*.

[11] Qu N., Qin S., Zhang X. (2020). Multiple m6A RNA methylation modulators promote the malignant progression of hepatocellular carcinoma and affect its clinical prognosis. *BMC Cancer*.

[12] Liu W., Zhong C., Lv D., Tang M., Xie F. (2020). N6-methyladenosine RNA methylation regulators have clinical prognostic values in hepatocellular carcinoma. *Frontiers in Genetics*.

[13] Fu Y., Liu S., Zeng S., Shen H. (2019). From bench to bed: the tumor immune microenvironment and current immunotherapeutic strategies for hepatocellular carcinoma. *Journal of Experimental & Clinical Cancer Research*.

[14] Kole C., Charalampakis N., Tsakatikas S. (2020). Immunotherapy for hepatocellular carcinoma: a 2021 update. *Cancers (Basel)*.

[15] Schizas D., Charalampakis N., Kole C. (2020). Immunotherapy for pancreatic cancer: a 2020 update. *Cancer Treatment Reviews*.

[16] Schizas D., Charalampakis N., Kole C. (2020). Immunotherapy for esophageal cancer: a 2019 update. *Immunotherapy*.

[17] Han D., Liu J., Chen C. (2019). Anti-tumour immunity controlled through mRNA m^6^A methylation and YTHDF1 in dendritic cells. *Nature*.

[18] Yi L., Wu G., Guo L., Zou X., Huang P. (2020). Comprehensive analysis of the PD-L1 and immune infiltrates of m^6^A RNA methylation regulators in head and neck squamous cell carcinoma. *Molecular Therapy--Nucleic Acids*.

[19] Niu Z. S., Niu X. J., Wang W. H. (2016). Genetic alterations in hepatocellular carcinoma: an update. *World Journal of Gastroenterology*.

[20] Rastogi A. (2018). Changing role of histopathology in the diagnosis and management of hepatocellular carcinoma. *World Journal of Gastroenterology*.

[21] Gu C., Shi X., Dang X. (2020). Identification of common genes and pathways in eight fibrosis diseases. *Frontiers in Genetics*.

[22] Gu C., Huang Z., Chen X. (2020). TEAD4 promotes tumor development in patients with lung adenocarcinoma via ERK signaling pathway. *Biochimica et Biophysica Acta - Molecular Basis of Disease*.

[23] Zhang L., Shi X., Gu C. (2020). Identification of cell-to-cell interactions by ligand-receptor pairs in human fetal heart. *Biochimica et Biophysica Acta - Molecular Basis of Disease*.

[24] Shi X., Shao X., Liu B. (2020). Genome-wide screening of functional long noncoding RNAs in the epicardial adipose tissues of atrial fibrillation. *Biochimica et Biophysica Acta - Molecular Basis of Disease*.

[25] Gu C., Shi X., Huang Z. (2020). A comprehensive study of construction and analysis of competitive endogenous RNA networks in lung adenocarcinoma. *Biochimica et Biophysica Acta, Proteins and Proteomics*.

[26] Deng X., Su R., Feng X., Wei M., Chen J. (2018). Role of _N_ ^6^-methyladenosine modification in cancer. *Current Opinion in Genetics & Development*.

[27] Gu C., Shi X., Qiu W. (2021). Comprehensive analysis of the prognostic role and mutational characteristics of m6A-related genes in lung squamous cell carcinoma.

[28] Lu J., Qian J., Yin S., Zhou L., Zheng S., Zhang W. (2020). Mechanisms of RNA N6-methyladenosine in hepatocellular carcinoma: from the perspectives of etiology. *Frontiers in Oncology*.

[29] Zhao X., Chen Y., Mao Q. (2018). Overexpression of YTHDF1 is associated with poor prognosis in patients with hepatocellular carcinoma. *Cancer Biomarkers*.

[30] Zhong L., Liao D., Zhang M. (2019). YTHDF2 suppresses cell proliferation and growth via destabilizing the EGFR mRNA in hepatocellular carcinoma. *Cancer Letters*.

[31] Tanabe A., Konno J., Tanikawa K., Sahara H. (2014). Transcriptional machinery of TNF-*α*-inducible YTH domain containing 2 (YTHDC2) gene. *Gene*.

[32] Chen M., Wei L., Law C. T. (2018). RNA N6-methyladenosine methyltransferase-like 3 promotes liver cancer progression through YTHDF2-dependent posttranscriptional silencing of SOCS2. *Hepatology*.

[33] Li L., Zheng Y. L., Jiang C. (2019). HN1L-mediated transcriptional axis AP-2*γ*/METTL13/TCF3-ZEB1 drives tumor growth and metastasis in hepatocellular carcinoma. *Cell Death and Differentiation*.

[34] Ma J. Z., Yang F., Zhou C. C. (2017). METTL14 suppresses the metastatic potential of hepatocellular carcinoma by modulatingN6‐methyladenosine‐dependent primary microRNA processing. *Hepatology*.

[35] Chen Y., Peng C., Chen J. (2019). WTAP facilitates progression of hepatocellular carcinoma via m6A-HuR-dependent epigenetic silencing of ETS1. *Molecular Cancer*.

[36] Li Z., Li F., Peng Y., Fang J., Zhou J. (2020). Identification of three m6A-related mRNAs signature and risk score for the prognostication of hepatocellular carcinoma. *Cancer Medicine*.

[37] Li W., Chen Q. F., Huang T., Shen L., Huang Z. L., Wu P. (2020). Profiles of m<sup>6</sup>A RNA methylation regulators for the prognosis of hepatocellular carcinoma. *Oncology Letters*.

[38] Chen L., Huang X., Zhang W. (2020). Correlation of PD-L1 and SOCS3 co-expression with the prognosis of hepatocellular carcinoma patients. *Journal of Cancer*.

[39] Tu S., Huang W., Huang C., Luo Z., Yan X. (2019). Contextual regulation of TGF-*β* signaling in liver cancer. *Cell*.

[40] Dimri M., Satyanarayana A. (2020). Molecular signaling pathways and therapeutic targets in hepatocellular carcinoma. *Cancers (Basel)*.

[41] Jeng K. S., Jeng C. J., Jeng W. J. (2019). Sonic Hedgehog signaling pathway as a potential target to inhibit the progression of hepatocellular carcinoma (Review). *Oncology Letters*.

[42] Su F., Zhao J., Qin S. (2017). Over-expression of thrombospondin 4 correlates with loss of miR-142 and contributes to migration and vascular invasion of advanced hepatocellular carcinoma. *Oncotarget*.

[43] Yu Q., Xiang L., Yin L., Liu X., Yang D., Zhou J. (2017). Loss-of-function of miR-142 by hypermethylation promotes TGF-beta-mediated tumour growth and metastasis in hepatocellular carcinoma. *Cell Proliferation*.

